# Antimicrobial stewardship programs in seven Latin American countries: facing the challenges

**DOI:** 10.1186/s12879-023-08398-3

**Published:** 2023-07-11

**Authors:** Christian José Pallares, Jessica Porras, Elsa De La Cadena, Juan Carlos García-Betancur, Natalia Restrepo-Arbeláez, Sara María Cobo Viveros, Wanda Cornistein, Paulo Castañeda-Méndez, Luis Cuellar, Diogo Boldim-Ferreira, Jorge Chaverri-Murillo, Jaime A. Labarca, María Virginia Villegas

**Affiliations:** 1 Grupo de Investigación en Resistencia Antimicrobiana y Epidemiología Hospitalaria (RAEH), Universidad El Bosque, Bogotá, Colombia; 2 Clínica Imbanaco, Grupo Quirónsalud, Cali, Colombia; 3 Hospital Universitario Austral, Buenos Aires, Argentina; 4 Department of Infectious Diseases, Hospital Médica Sur, Ciudad De México, México; 5 Servicio de Infectología, Instituto Nacional de Enfermedades Neoplasicas, Lima, Perú; 6 Division of Infection Control and Hospital Epidemiology, Hospital São Paulo, Universidade Federal de São Paulo, São Paulo, Brazil; 7 Hospital Rafael Ángel Calderón Guardia, San José, Costa Rica; 8 Department of Infectious Diseases, School of Medicine, Pontificia Universidad Católica de Chile, Santiago de Chile, Chile

**Keywords:** Antimicrobial stewardship, Antimicrobial stewardship programs, Latin America, Antimicrobial resistance

## Abstract

**Background:**

Studies have shown that more than 50% of the antibiotics used in hospitals are unnecessary or inappropriate and, that antimicrobial resistance may cost up to 20 billion USD in excess medical costs each year. On the other hand, Antimicrobial Stewardship Programs (ASP) significantly reduce inappropriate antimicrobial use, emergence of antimicrobial resistance, healthcare associated infections, and costs in hospital settings.

**Objective:**

To evaluate the development of ASP and antibiotic savings in 7 Latin American hospitals using standardized quantitative indicators in all the participating health care institutions.

**Methods:**

An interventional study was conducted, where pre- and post- evaluations were performed using a standardized score tool adapted from the Joint Commission International accreditation standards and, the Colombian Institute of Technical Standards and Certification. We evaluated ASP from 7 Latin American hospitals between 2019 and 2020. A *pre-intervention* evaluation was done in each hospital to quantify the degree of development of the ASP (ASP Development score). Based on these results, tailored on-site training was implemented in each hospital, followed by a *post-intervention* evaluation to quantify improvement of ASP-development indicators. In addition, monetary savings in antimicrobials derived from the ASP intervention were estimated.

**Results:**

In the pre-intervention evaluation, the average ASP development score for the 7 institutions was 65.8% (40-94.3%). The items with the lowest development score were those related to monitoring and communicating the ASP progress and success. For the post-intervention evaluation, 2 institutions couldn’t participate due to the pressure imposed by the COVID-19 pandemic. For the remaining 5/7 hospitals, the average ASP development score was 82.3% with an increase of 12.0% when compared to the pre-intervention measurement of the same institutions (average pre-intervention score 70.3% (48.2%-94.3%) The items with a significant increase were key performance indicators, AMS education and training of the prescribers. Three of the seven (3/7) hospitals reported antibiotic monetary savings associated to the ASP intervention.

**Conclusions:**

The use of the tool described shown to be useful to evaluate specific areas of ASP-development that were lacking and tailor interventions for the participating hospitals, consequently, it helped improve ASP-development in the institutions that underwent pre- intervention and post-intervention analysis. In addition, the strategies showed monetary savings on antimicrobial costs when measured.

**Supplementary Information:**

The online version contains supplementary material available at 10.1186/s12879-023-08398-3.

## Background

In last decades, antimicrobial resistance (AMR) has increased exponentially, becoming a public health problem, with an important impact on healthcare costs [1, 2]. Only in the United States, the costs associated to AMR is around 55 billion USD every year [3]. Also, the World Bank reports that AMR increases the rate of poverty with a higher impact on low-income countries (LICs), calculating a decrease in the annual Gross domestic product (GDP) of approximately 5–7% [3]. In response, national plans to tackle AMR are being implemented in several countries [4–6]. Briefly, the general framework of an Antimicrobial Stewardship Program (ASP) includes: (a) system prerequisites (i.e., structure of an ASP) that must be achieved before its implementation (e.g., antimicrobial guidelines development, the conformation of the ASP team, adequate microbiology diagnostics, human and technological resources), (b) Clearly defined goals of what the ASP intends to improve (i.e., the processes that need to be addressed, duration of treatment and adherence to guidelines), and finally, (c) planning of how the ASP team will achieve these goals, the strategies that will be chosen and tailored based on the identified goals (e.g., audit and feedback, education) [7, 8]. ASP is a coherent set of actions which promote responsible antibiotic use [8], reduce AMR and decrease the spread of multidrug-resistant (MDR) organisms [9, 10]. Through ASPs, the patient should get the right drug in the right dose and with the right duration, including de-escalation from the initial broad-spectrum antibiotics to a narrow spectrum when the patient is clinically stable and a culture is reported [9].

For the successful implementation of an ASP, close interdisciplinary collaboration is required among different healthcare professionals, including but not restricted to the infectious disease physicians, nurses, pharmacists, often an infection preventionist or epidemiologist, and the microbiologist. This interdisciplinary work also integrates the administrative staff and the hospital manager [9, 11, 12].

ASPs have been increasingly and successfully implemented around the world, particularly in high-income countries (HICs) such as the United States, United Kingdom or France [13, 14]. However, less has been done, and few is known about ASP in low- and middle-income countries (LMICs) [13, 15]. In particular, Latin America (LATAM) has a relatively low number of indexed publications on the evaluation of clinical, pharmacological, or microbiological aspects of ASPs [9], and has focused primarily on educational interventions followed by persuasive and restrictive antimicrobial strategies [13].

In this study, we report the evaluation of ASP-development and implementation of interventions for the optimization of ASPs in 7 LATAM hospitals, as well as, monetary savings of antibiotics (through suspension, change, descaling, or time-reduction of therapy), using a quantitative standardized tool adapted from the Colombian Institute of Technical Standards and Certification (ICONTEC), as well as previous consensus of national experts and the Joint Commission International (JCI) accreditation standards) [16–18].

## Methods

A pre- and post-intervention study was conducted. Seven LATAM hospitals were selected in the study. They were chosen based on its recognition as Excellence Centers for AMS (Antimicrobial Stewardship) in the region (high-complexity medical institutions able to develop a national ASP, positive leadership, with inter-institutional, national and / or government recognition, skills in communicating and transferring scientific knowledge, and motivational management). We selected one hospital per country in Argentina, Brazil, Chile, Colombia, Costa Rica, Mexico, and Peru. **Supplementary Table** 1 displays basic information about the participating hospitals, including level of healthcare attention and capacity. The initial contact with each hospital was made through the infectious disease specialist running the ASP. Once confirmation for participation was obtained from the hospital director, a series of face-to-face visits and teleconferences were carried out in a 20-month period.

The study consisted of 2 phases. In phase-1, a pre-intervention evaluation was carried out using a quantitative standardized tool that was adapted from ICONTEC, and JCI accreditation standards, as well as supported by a previous consensus of national experts [16–18]. Specifically, this tool in **Table** 1, consisted of a questionnaire, where each participant hospital should answer in a binary mode (Yes/No), giving every positive (Yes) response a partial percentage within each category (standard). Each standard was given an average score based on the evaluated items using percentages, and later, the proportional weight of each Standard was added to calculate the end-result. This tool quantified 7 Standards of the development of the ASP in each selected hospital. The results for each Standard evaluated were reported in 4 categories: high (H) = 100%, medium (M) 66%, low (L) 33% or, none (N) 0%.

Based on this pre-intervention evaluation, phase-2 of the study was implemented. This phase consisted of a series of customized interventions suggested to each participating hospital, based on training and capacity-building strategies within the ASPs where the standardized tool identified lowest development. It included: a written document establishing the creation of a formal ASP accepted by the hospital administration, the incorporation of the infection prevention and control committee to the ASP, the implementation and/or evaluation of cleaning and disinfection protocols, the creation and use of epidemiological reports utilizing the World Health Organization network tool (WHONET) for AMR monitoring. The creation and/or implementation of updated antimicrobial guidelines, cost savings when inappropriate antibiotics were stopped and/or changed by the ASP team, as well as individual outcomes for each hospital. Face-to-face, and then virtual meetings were carried out in each hospital, discussing each recommendation for the implementation and/or improvement of the ASPs with each hospital team. Virtual monitoring meetings were held every month to evaluate the development of the implementation, as well as discussion of new challenges faced, to be solved among the teams, and periodic outcomes reported. After implementation of the ASP strategies and their development, the post-intervention evaluation was done with the same standardized tool. Post-intervention evaluation could be applied only to 5/7 institutions because the Brazilian and the Costa Rican hospitals had to abandon the post-intervention evaluation due to the COVID-19 pandemic, mainly related to the redistribution of the AMS team to manage the high demand of healthcare professionals. The degree of change in ASP-development, improvement achieved by these interventions and the subsequent monitoring meetings were measured by comparing the reported percentages (scores) of development (H, M, L or N) before and after implementation of the interventions. For the calculation of monetary cost savings, the cost of the patient’s antibiotic formulation before the ASP intervention was subtracted from the cost of the new therapy recommended by the ASP. The interventions considered were stopping the initial treatment, de-escalating to another antibiotic, shortening the time of therapy, or changing the antimicrobial from intravenous to oral. The final economic result of the ASP interventions was the difference on antimicrobial cost, and implementation costs between the two therapies.

**Table 1:** **Evaluation tool used in the participating healthcare institutions for the phases 1 and 2 progress of their ASP**

Standard		Measurable elements
**1**	Hospital leadership support (3 items/ weight: 5%)	*1) The AMS is part of the institutional goals in the hospital of senior management.* *2) There is an institutional budget for the AMS.* *3) Hospital managers know the national or international AMS standards.*
**2**	Assemble an AMS team structure (5 items/ weight: 15%)	*1) The AMS has a written and related policy in official documents.* *2) The AMS has human talent organized under a structure (committee, program, area, or section within the hospital).* *3) The members of the AMS have a defined position within the institutional human talent area* *4) The AMS has representatives from different hospital areas (surgery, internal medicine, intensive care, emergencies) within the institution.* *5) The AMS has standard operating procedures that regulate its operation in the hospital.*
**3**	AMS core team member roles and responsibilities (5 items / weight: 20%)	*1) The microbiology laboratory is articulated with the infection and epidemiological surveillance committee to obtain microbiology and laboratory reports from patients with suspected / diagnosed nosocomial infection.* *2) The infection committee conducts prospective active surveillance of health care-associated infections.* *3) The infection committee actively monitors hand hygiene, use of personal protection elements and standard and specific precautions (contact, air, drops, protector).* *4) The institution has standardized processes for cleaning and disinfection of the hospital environment (including surveillance).* *5) The institution has standardized processes for cleaning and disinfecting of biomedical equipment and devices (including surveillance).*
**4**	AMS education and training (2 items/ weight: 10%)	*1) Physicians receive training courses in antibiotic therapy.* *2) AMS members receive formal training in antimicrobial resistance and therapy.*
**5**	Selecting goals (3 items/ weight: 20%)	*1) The AMS has members guiding / advising the decisions of the physicians.* *2) There is a list of drugs approved by the AMS team for its use (control of spending).* *3) The institution has a guideline for the use of antibiotics based on updated local epidemiology and in consensus with the medical specialties.*
**6**	Selecting and monitoring key performance indicators (3 items/ weight: 20%)	*1) The institution carries out active surveillance of the prescription of antibiotics in the institution (outcomes indicators).* *2) There is measurement of antimicrobial consumption in the institution* *3) The institution has an updated microbiological profile of antimicrobial resistance.*
**7**	Monitor and communicate AMS program progress and success (2 items/ weight: 10%)	*1) The institution makes periodic reports about the findings of the AMS surveillance.* *2) The institution socializes the findings of the AMS surveillance with prescribers.*

Based on: [5, 16–18]

## Results

Results from Phase 1 (pre-intervention) and Phase 2 (post-intervention) evaluations from the 7 participating hospitals in LATAM countries are summarized in **Table** 2.

**Table 2:** **Pre- and post-intervention ASP Standard evaluation for 7 LATAM hospitals between 2019 and 2020**

Hospital country	ARGENTINA				BRAZIL			CHILE					COLOMBIA			COSTA RICA				MEXICO			PERU		
Standard	Pre	Post	Improvement (%)		Pre	Post	Improvement (%)	Pre	Post		Improvement (%)		Pre	Post	Improvement (%)	Pre	Post		Improvement (%)	Pre	Post	Improvement (%)	Pre	Post	Improvement (%)
**1**	88,7	88,7	**0,0**		33,0	*	N/A	66,3	66,3		**0,0**		100,0	100,0	**0,0**	11,0	*		N/A	100,0	100,0	**0,0**	0,0	33,3	**100,0**
**2**	86,6	93,2	**7,1**		79,8	*	N/A	33,0	66,0		**50,0**		100,0	100,0	**0,0**	46,4	*		N/A	93,2	100,0	**6,8**	66,2	66,2	**0,0**
**3**	73,0	79,6	**8,3**		73,2	*	N/A	86,4	86,4		**0,0**		100,0	100,0	**0,0**	73,0	*		N/A	66,0	66,0	**0,0**	66,4	66,4	**0,0**
**4**	49,5	100,0	**50,5**		66,0	*	N/A	49,5	49,5		**0,0**		100,0	100,0	**0,0**	50,0	*		N/A	100,0	100,0	**0,0**	33,0	100,0	**67,0**
**5**	88,7	100,0	**11,3**		100,0	*	N/A	77,7	77,7		**0,0**		100,0	100,0	**0,0**	55,3	*		N/A	55,3	55,3	**0,0**	66,7	100,0	**33,3**
**6**	33,0	77,7	**57,5**		66,3	*	N/A	44,0	44,0		**0,0**		77,3	100,0	**22,7**	44,3	*		N/A	88,7	100,0	**11,3**	55,3	100,0	**44,7**
**7**	83,0	83,0	**0,0**		66,0	*	N/A	33,0	33,0		**0,0**		83,0	100,0	**17,0**	0,0	*		N/A	66,0	100,0	**34,0**	49,5	49,5	**0,0**
**IMPROVEMENT AVERAGE**		**ARGENTINA**				**BRAZIL**			**CHILE**				**COLOMBIA**			**COSTA RICA**				**MEXICO**			**PERU**		
		**Pre**	**Post**	**Improvement (%)**		**Pre**	**Post**	**Improvement (%)**	**Pre**	**Post**		**Improvement (%)**	**Pre**	**Post**	**Improvement (%)**	**Pre**	**Post**	**Improvement (%)**		**Pre**	**Post**	**Improvement (%)**	**Pre**	**Post**	**Improvement (%)**
		**71,8**	**88,9**	**17,1**		**69,2**	*	N/A	**55,7**	**60,4**		**4,7**	**94,3**	**100,0**	**5,7**	**40,0**	*	N/A		**81,3**	**88,8**	**7,5**	**48,2**	**73,6**	**25,5**

Pre: Pre intervention evaluation, Post: Post-intervention evaluation, * due to COVID-19, these data could not be retrieved

### Pre-intervention evaluation

According to the diagnostic tool implemented in 7/7 hospitals, the average ASP-development score was 65.8% (40.0 − 94.3%). In summary, Costa Rica (40.0%) showed the lowest development score, followed by Peru (48.2%) and Chile (55.7%). On the other hand, Colombia showed the highest ASP development with a 94.3%, followed by Mexico (81.3%), Argentina (71.8%) and Brazil (69.2%).

The standards with the least institutional development before the intervention were those related to Monitor and Communicate AMS Program progress and success (54.4%) and Hospital Leadership support (57.0%). On the other hand, the Standards with the highest development were those related to Selecting goals (77.7%) and ASM core team member roles and responsibilities (76.9%).

### Post-intervention evaluation

In the post-intervention phase, the Brazilian and Costa Rican hospitals could not continue participation in the study because of the COVID- 19 pandemic, which stopped most ASP activities. The average score for the remaining 5/7 hospitals was 82.3%, achieving an average increase of 12.0% (5.7-35.0%), when compared with the same 5 hospital’s score during the pre-intervention phase 70.3%  (48.2%-94.3%). Peru was the country with the most improvement after the intervention (25.5%), obtaining a final score of 73.6%, improvement was especially perceived in (Standard 1) Hospital leadership support, (Standard 4) AMS education and training, (Standard 5) Selecting goals, and (Standard 6) Selecting and monitoring key performance indicators. Colombia was the country with the highest achieved score (100%), improved performance was achieved in (Standard 7) Monitor and communicate AMS program progress and success and Selecting and Monitoring key performance indicators. Argentina achieved the second highest score (88.9%), with improvement especially in AMS education and training and Selecting and monitoring key performance indicators.

At the end of the study period, the Standard items with the greatest phase-2 (post-intervention) increase were Selecting and Monitoring key performance indicators (up to 27.2%) and AMS education and training (up to 23.5%) as seen in **Table** 2.

### Monetary antibiotics savings (economic impact measurement)

Only 3/7 hospitals reported antibiotics savings related to the implemented ASP interventions: Mexico with a net saving of USD 42,061/month, Colombia with a net saving of USD 26,453/month and Argentina with a net saving of USD 3,761/month. The remaining 4 hospitals couldn’t estimate economic impact results. Participating institutions in Brazil and Costa Rica could not continue evaluation because of ASP team disintegration during the COVID-19 pandemic, while participating hospitals in Chile and Peru could not obtain data due to administrative restrictions of the clinical ASP team’s access to billing data on antibiotic use and invoicing.

## Discussion

According to the Pan American Health Organization (PAHO), 30 of 33 countries in Latin America and the Caribbean are in the process of developing their national action plans to fight AMR [19]. However, only 19 of the 33 countries report to the Latin American Network for Antimicrobial Resistance Surveillance leaving a gap regarding this information [20].

In 2015, a survey assessing the development of ASPs in 27 hospitals in 10 countries throughout Central and South America [21], reported that only 59.0% of the hospitals had a formal written statement supporting the creation of the ASP (16 out of 27). In the same survey, only nine of the 27 hospitals (33.3%) had Antimicrobial Guidelines based on their local epidemiology and only 48.0% performed prospective audit and feedback [20, 21].

In our study, despite the clear increase in ASP development, for (Standard 1) Hospital leadership support, it is still key that every hospital has a formal statement of creation and funding for their AMS activities from the hospital management [22]. Although this is a crucial step, it may not be considered a priority as it competes for resources with other hospital needs. It is therefore important to provide hospital management with a credible business case and cost-utility value, to persuade them that funding for an ASP is beneficial to the hospital.

The Standard item with the greatest average increase was Selecting and Monitoring key performance indicators, which was 59.6% in the pre-intervention period and increased after the intervention to 84.4%. Antimicrobial Guidelines based on local data surveillance are key in the ASP implementation because their compliance leads to quality improvement, and better patient outcomes [23]. Finally, Monitoring and Communicating the ASM program progress and success was also lacking, with a score of 63% during pre-intervention evaluation. ASP teams should provide ongoing training and feedback sessions that emphasize the purpose and importance of ASPs, as well as communicating all positive outcomes that are occurring in association with the hospital ASP [5, 16]. Education on ASP and details of the hospital own ASP should be routinely provided as part of the training for new staff, with regular updates to keep all staff informed about any changes related to the ASP procedures and goals; without showing the successful results, it will be difficult to have visibility among the healthcare professional and increase the administration support needed to obtain more resources, as well as to demonstrate the impact of the ASP in certain areas or hospital wide [22].

As described in some articles, the low level of political commitment, the scarcity of ASP funding as well as the dearth of expertise in orchestrating ASP initiatives, are some of the biggest hurdles to ASP adoption among low- and middle- income countries [15, 24]. During the study, it was remarkably noticed that specific funding, and organizational structures supported by leadership were essential for ASP activity completion and sustainability of the program. Commitment of hospital leadership is also achieved by clear communication of activities and progress of AMS progress, because if there is no knowledge of the strategies from the ‘Top-down’, ASPs won’t improve their development.

Lastly, several studies have shown that ASPs are cost-effective [20]. A systematic literature review showed at least 15 studies, reporting antibiotic cost savings; on average, the antibiotic costs after the ASP interventions were $1630 USD per 100 patient-days compared to $2078 USD per 100 patient-days in the controls. This corresponds to an average cost reduction of $448 USD per 100 patient- days or 25%, respectively [9]. On the other hand, in our study, Mexico had a net saving from antibiotic and implementation of therapy costs of 42,061USD per month, Colombia 26,453 USD per month, and Argentina 3,761 USD per month. This indicator should be considered by administrators to support ASPs in LATAM and reiterate the value for hospital management.

The study’s principal strengths are the fact that is the first study from our knowledge in LATAM to seek and utilize a standardized quantitative tool, to generate a metric score for the evaluation of ASP development. In contrast, the main limitations were the withdrawal of 2/7 participating healthcare institutions from the post-intervention analysis due to the COVID-19 pandemic, a reality faced by many hospitals in the world; the other limitation was that even if it was possible to stablish the level of development in ASP, changes in the human resources, as well as changes of stakeholders (private/public insurers, state) that fund healthcare provision and in turn ASPs, could have influenced the results.

Future studies should focus on ASP results regarding infections due to specific antimicrobial-resistant pathogens, detail cost savings between appropriate vs. inappropriate antibiotics, and mortality related to the lack of ASP; all this effort should help increase the visibility of ASP in LATAM.

## Conclusions

The challenges for implementation of ASP interventions are many, and the published evidence for the effectiveness of stewardship interventions in LMICs is limited. However, in our study, the use of a quantitative tool to measure the improvement before and after the ASP implementation was extremely useful to evaluate the level of ASP development, allowing to focus the intervention on specific areas with the lowest development. Quantitative tools may help improve the development of ASPs in all the hospitals as was seen in our study, guiding intervention implementation, and post-intervention analysis, showing improvement of ASP standards and success. We do believe that it is possible to have strong ASP in all LATAM countries, but more government and leadership support is needed, especially funding allocated for ASP activities in particular to guarantee sustainability of the programs.

## Electronic supplementary material

Below is the link to the electronic supplementary material.

Supplementary Material 1

## Data Availability

All data generated or analyzed during this study are included in this published article.

## List of abbreviations

AMS: Antimicrobial Stewardship.
ASP: Antimicrobial Stewardship Program.
GDP: Gross domestic product.
HIC: High-income countries.
ICONTEC: Instituto Colombiano de Normas Técnicas y Certificación.
JCI: Joint Commission International.
LATAM: Latin America.
LIC: Low-income countries.
LMIC: Low- and middle-income countries.
MDR: Multi-drug resistant.
PAHO: Pan American Health Organization.
WHONET: World Health Organization Network Tool.
