# Supplementary material for: Antimicrobial stewardship programs in seven Latin American countries: facing the challenges
Source: BMC Infect Dis. 2023 Jul 11;23:463. doi: 10.1186/s12879-023-08398-3 (PMC10334602; doi:10.1186/s12879-023-08398-3)
Supplement: Supplementary file 1 — Supplementary Material 1 [file 12879_2023_8398_MOESM1_ESM.docx]

**Supplementary Material**

**Supplementary Table 1:** basic information about the participating hospitals in 7 LATAM countries.

| **Country** | **City** | **Teaching hospital** | **Hospital beds (total)** | **Hospital beds (ICU)** | **Attention level**  **(1,2,3,4)** | **Surgeries per year** |
| --- | --- | --- | --- | --- | --- | --- |
| **Argentina** | Buenos Aires | Yes | 220 | 21 | 4 | 20.000 |
| **Brazil** | São Paulo | Yes | 501 | 126 | 3 | 14.640 |
| **Chile** | Santiago | Yes | 420 | 72 | 4 | 12.000 |
| **Colombia** | Cali | No | 257 | 64 | 4 | 25.000 |
| **Costa Rica** | San José | Yes | 498 | 28 | 3 | 20.000 |
| **Mexico** | Mexico D.F. | Yes | 189 | 38 | 3 | 11.771 |
| **Peru** | Lima | Yes | 402 | 18 | 3 | 7.000 |
